# Regulatory T Cells Improved the Anti-cirrhosis Activity of Human Amniotic Mesenchymal Stem Cell in the Liver by Regulating the TGF-β-Indoleamine 2,3-Dioxygenase Signaling

**DOI:** 10.3389/fcell.2021.737825

**Published:** 2021-10-12

**Authors:** Zhenhua Deng, Jinren Zhou, Xiaoxin Mu, Jian Gu, Xiangyu Li, Qing Shao, Jinyang Li, Chao Yang, Guoyong Han, Jie Zhao, Yongxiang Xia

**Affiliations:** Hepatobiliary Center, The First Affiliated Hospital of Nanjing Medical University, Liver Cancer Institute, Nanjing Medical University, Nanjing, China

**Keywords:** liver cirrhosis, hAMSC, Treg, IDO, TGF-β

## Abstract

Liver fibrosis is a progression stage of chronic liver disease, while current therapies cannot cure or attune cirrhosis effectively. Human amniotic mesenchymal stromal cell (hAMSC) presented immunoregulatory and tissue repairability of multiple illnesses. Regulatory T cells (Treg) had been proved to be functional in reducing immune cell activity. We showed that co-infusion of hAMSC and Treg prevented mild liver fibrosis comparing with hAMSC or Treg alone group. *In vitro* study indicated that the addition of Treg or the supernatant of Treg improved the hepatocyte growth factor (HGF) secreting and cell differentiation ability of hAMSC. Reduction of TGF-β significantly decreased the HGF secreting and differentiation of hAMSC. Multiple signal neutralizers were added to the culture to understand further the mechanism, which showed that 1-MT, the suppressor of Indoleamine 2,3-dioxygenase (IDO), was involved in the effect of TGF-β in regulating hAMSC. Depletion of TGF-β or IDO signaling successfully abolished the effect of Treg in improving hAMSC’s function both *in vitro* and vivo. Finally, our result indicated that Treg improved the function of hAMSC by regulating the TGF-β-IDO signaling and co-infusion of hAMSC and Treg provided a promising approach for treating liver cirrhosis.

## Introduction

Liver cirrhosis is unrecoverable progress that may cause liver failure or liver cancer in chronic liver disease. Histopathologically, extensive necrosis of liver cells, nodular regeneration of residual liver cells, connective tissue hyperplasia, and fibrous septum formation are the main features of cirrhosis (Tsochatzis et al., 2014).

Human amniotic mesenchymal stem cell (hAMSC) is isolated from the amniotic membrane (AM) of the full-term human placenta (Li et al., 2020). It has similar characteristics with typical MSC, including fibroblastic morphology, specific surface molecules, and multidirectional differentiation potential. In addition, it has excellent immunomodulatory and paracrine properties (Magatti et al., 2015, 2018; Silini et al., 2017). Human amniotic mesenchymal stem cell shows low immunogenicity because it does not express human major histocompatibility complex (MHC) or antigens (human leukocyte antigen, HLA) (Evangelista et al., 2008).

CD4^+^ Foxp3^+^ regulatory T cell (Treg), a subset of CD4^+^ T cells, plays a key role in maintaining immune tolerance and immune homeostasis in different tissues. It is mainly composed of natural Treg cell (tTreg cell) and induced Treg cell (pTreg cell) (Yang et al., 2021). Foxp3 is an important marker of Treg cell and a major carrier of differentiation and function of Treg cell. Low expression of Foxp3 leads to the ablation of Treg, which results in inflammatory disease in mice or humans.

Transplantation of hAMSC has been reported to improve liver function in animal models (Kubo et al., 2015; Zhang et al., 2011). However, we found that the therapeutic effect of hAMSC infusion alone in mice with cirrhosis was modest and inconsistent. To improve the therapeutic effect, we tried to adoptive transferring Treg together with hAMSCs into cirrhosis mice. The results showed that the liver function and liver lesions of mice were significantly improved. Mechanism study proved that Treg expressed TGF-β, which activated the Indoleamine 2,3-dioxygenase (IDO) signal of hAMSC and improved hepatocyte growth factor (HGF) secreting of hAMSC. Taken together, our data presented a novel therapeutic strategy of anti-cirrhosis in chronic liver disease.

## Materials and Methods

### Animals

Male mice (C57BL/6) were purchased from Nanjing Medical University. Mice at the age of 6–8 weeks were used. We maintained breeding colonies in our SPF (Specific Pathogen Free) facility. All animals received humane care according to the guidelines for experimental animals and were approved by the Institutional Animal Care and Research Advisory Committee of Nanjing Medical University.

### Differentiation of Human Amniotic Mesenchymal Stem Cell

#### Adipogenic Differentiation

Stem cells were cultured in a 5% CO2 incubator at 37°C. When the cell fusion degree reached 80% to 90%, Stem cells were digested with the TrypLE enzyme. Then the digested stem cells were inoculated in a six-well plate and a 2 mL complete medium was added to each well. After that, the cells were cultured in the incubator of 5% CO2 at 37°C again. Change the fluid every 2–3 days until the cell fusion reaches 100% or overfuses. The growth medium was abandoned and 2 mL PADM (Sciencell 7221) was added to the six-well plate and PADM was changed every 2–3 days. Stem cells were induced for 21–30 days until the lipid droplets became large and round enough and Oil red O staining was performed.

#### Osteogenesis Differentiation

Stem cells were cultured in a 5% CO2 incubator at 37°C. When the cell fusion degree reached 80% to 90%, Stem cells were digested with the TrypLE enzyme. Then the digested stem cells were inoculated in a six-well plate and a 2 mL complete medium was added to each well. After that, the cells were cultured in the incubator of 5% CO2 at 37°C again. Change the fluid every 2–3 days until the cell fusion reaches 100% or overfuses. The growth medium was abandoned and 2 mL MODM (Sciencell 05465) was added to the six-well plate and PADM was changed every 3–4 days. After 21 days of induction, the cells were stained with alizarin red and monitored according to the morphological changes and the growth of the cells.

### Cultivation of the Human Amniotic Mesenchymal Stem Cell

Human amniotic mesenchymal stem cells (hAMSCs) was provided by the Stem Cell Clinical Trial and Research Base of Jiangsu Provincial People’s Hospital. The cells were precipitated by centrifugation and resuspended in DMEM/F12 medium containing 10% FBS. The cells were inoculated in a culture flask and cultured in an incubator with 5% CO2 at 37°C. After cultivation for 48 h, replace the culture medium, discard the non-adherent cells and change the culture medium every 2 or 3 days. Add trypsin to digest and observe the cell digestion with an inverted phase-contrast microscope when the cells grow to 80% to 90%. And the cells were subcultured according to the proportion of 1:3.

### Co-culture of Tregs and Human Amniotic Mesenchymal Stem Cells *in vitro*

For contact-independent co-culture, Treg and hAMSC were cultured and physically separated using a 0.4-μm porous transwell system. Treg was seeded in the transwell nest and hAMSC was placed in the lower chamber (12-well transwell plate). The ratio between hAMSC and Treg was 1:4. After 72 h, the supernatant of the co-culture system for cytokine measurement was collected. Some experiments added anti-TGF-β monoclonal antibody (0.5 μg/ml) and anti-IL-10 monoclonal antibody (0.5 μg/ml) at the beginning of co-culture. Besides, after co-culture, hAMSC of the lower chamber was collected for injection.

### Mouse Model of Liver Fibrosis and Cirrhosis

The 10% volume fraction of carbon tetrachloride was dissolved in olive oil, and the bodyweight of the mice was measured. The mice were injected intraperitoneally according to the dose of 10 μl/g, and the injection frequency was maintained at 3 times per week. In addition, mice were executed at week 4 and week 8 to monitor the progression of liver fibrosis and used after 12 weeks.

### Cell Injection

Cells were injected 12–24 hours earlier in beginning of liver fibrosis modeling. Treg and AMSC were mixed and injected into mice through the tail vein. The ratio of of Treg and hAMSC was 1:4. Treg (10^6^/30 g) and hAMSC (0.25 × 10^6^/30 g) were suspended in 200 μl PBS and then injected into mice. In some experiments, Treg was co-cultured with hAMSC in a transwell chamber. After 3 days, the lower layer hAMSC was digested and counted, then re-suspended in 200 μl PBS, and injected into mice via caudal vein according to the dose of 0.25 × 10^6^/30 g. The sham group of all experiments were only injected with 200 μl PBS.

### Chemical and Histological Assessment of Liver Injury

Blood samples were collected from the eyes of mice with liver fibrosis. After centrifugation at 12000r for 10 min, the supernatant was measured for ALT, AST, and total bilirubin. The liver tissue was fixed with formalin and then embedded in paraffin. The specimen was cut into 4μm thick, then baked, soaked in xylene, and dehydrated with different concentrations of ethanol. After hematoxylin-eosin (HE) staining, the sections were placed under a light microscope for pathological analysis by the pathologist.

### Treg Acquisition

Suspensions of murine leukocytes were obtained from mice lymph nodes and spleens. Naïve CD4^+^ T cells were then acquired by auto-MACS (Miltenyi, San Diego, CA, United States) according to their CD4^+^/CD62L^+^ surface marker. Then, naïve T cells were activated in 96-well plates with completed media supplemented with anti-CD3/28 beads (Dynal beads, 1:1). For the differentiation of Treg, cells were cultured in the presence of IL-2 (100 U/ml, R&D Systems), TGF-β (5 ng/ml, R&D Systems), and anti-CD3/CD28 beads (Cell: beads = 2:1). The completed media is RPMI supplemented with 10% heat – inactivated FBS.

### Cytokine Measurement

HGF and stromal cell-derived factor 1 (SDF-1) levels were measured using enzyme-linked immunosorbent assay (ELISA) kits (BioLegend, United States). The supernatant to be detected was added to the orifice plate pre-coated with antibody and incubated for 30 min. The excess samples were washed off, and another primary antibody specific to the test was added and incubated for 30 min. The excess unbound primary antibody was washed off, and the secondary antibody with enzyme was added and incubated for 30 min. The excess unbound secondary antibody was washed off, the enzyme-substrate was added, and the OD value was measured at 450 nm wavelength.

### Statistical Analysis

All data were presented as the mean ± SD from at least three independent experiments. Statistical analysis was performed by the Student’s *t* test, one-way analysis of variance using GraphPad Prism8.0 software. Probability (P) values ≤ 0.05 were considered statistically significant. ^∗^, *p* < 0.05; ^∗∗^, *p* < 0.01; ^∗∗∗^, *p* < 0.001.

## Results

### Co-transfer of Treg Treatment Enhanced the Anti-cirrhosis Function of Human Amniotic Mesenchymal Stem Cell

**FIGURE 1:**
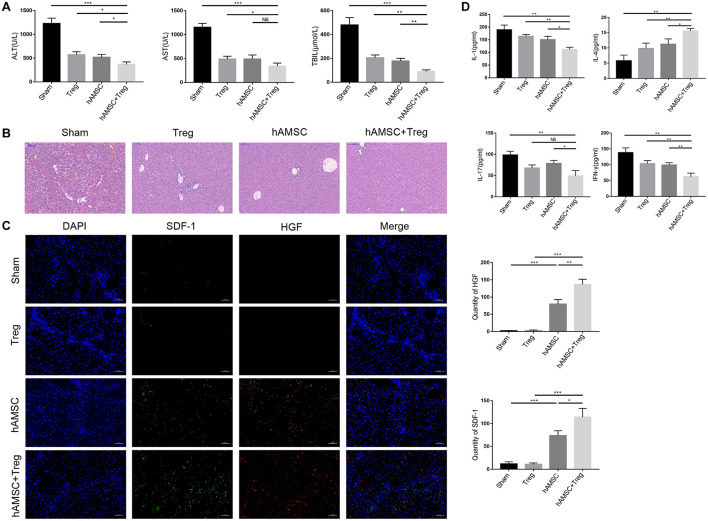
PBS, Treg, hAMSC, and hAMSC + Treg were injected into mice through the tail vein, respectively. **(A)** Level of ALT, AST, and TBIL of four groups. **(B)** Representative images of liver tissue [hematoxylin and eosin (HE) staining] by light microscopy in four groups. **(C)** Representative fluorescent images for SDF-1 and HGF in four groups were visualized by fluorescence microscopy, and DAPI was used to mark nuclei. **(D)** Level of IL-1, IL-4, IL-17, and IFN-γ in sham group, Treg group, hAMSC group, and cotransfer group. The result is representative of three independent experiments. Data were mean ± SD of three independent experiments. * *p* < 0.05, ** *p* < 0.01, *** *p* < 0.001.

We set up four groups (n = 4/group) to evaluate the effect of hAMSC or Treg on cirrhosis in mice. PBS, Treg, hAMSC, and hAMSC + Treg were injected into mice through the tail vein, respectively. The results showed that Treg, hAMSC, and co-transfer group presented effective anti-cirrhosis function, but the co-transfer group is more functional than any other group. Co-transfer of Treg and hAMSC showed lower ALT, AST, and TBIL (Figure 1A) and better fibrosis (Figure 1B) level compared with the other three groups. HGF and SDF-1 (Stromal cell-derived factor 1) are important liver proteins that have been proved to be expressed by MSC and participated in liver tissue repair (Kim et al., 2014; Wu et al., 2017; Ding et al., 2018; Jin et al., 2018; Mayorga et al., 2018). We used Immunofluorescence and confocal microscopy to observe the expression of SDF-1 and HGF in four groups of liver cirrhosis. It was found that co-transfer of Treg and hAMSC could secrete the most HGF and SDF-1 comparing with hAMSC or Treg alone group, thus limited the damage and reverted the cirrhosis of the liver (Figure 1C). Inflammatory factors play a fundamental role in liver cirrhosis. We also tested the expression of cytokines in the liver, which showed that co-transfer of Treg and AMSC most effectively reduced the pro-inflammatory cytokine expression such as IL-1, IL-17, IFN-γ and increased expression of IL-4, comparing with hAMSC group (Figure 1D).

### Treg Enhanced the Differentiation and Paracrine Function of Human Amniotic Mesenchymal Stem Cell *in vitro*

**FIGURE 2:**
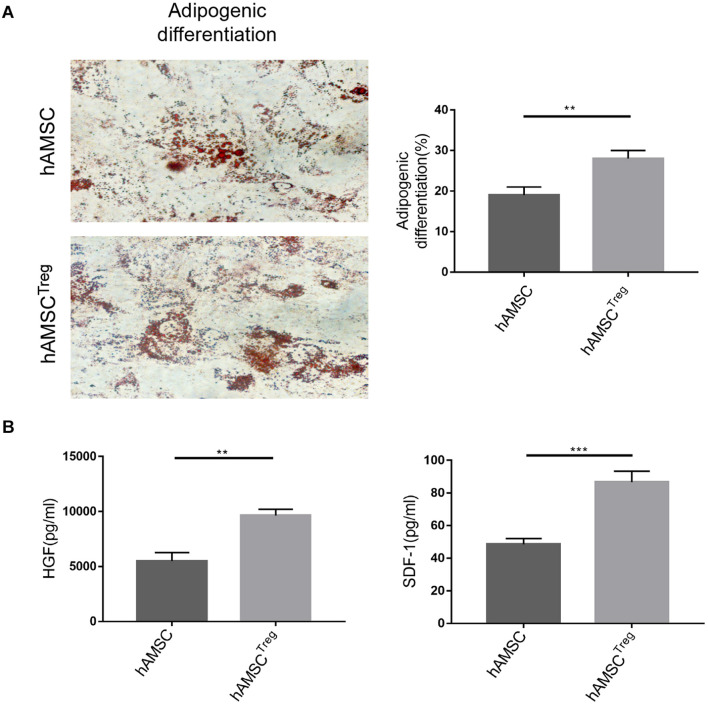
hAMSC was treated with PBS and Treg *in vitro*. **(A)** Representative images of hAMSC’s adipogenic differentiation in two groups. **(B)** Level of HGF and SDF-1 in two groups. The result is representative of three independent experiments. Data were mean ± SD of three independent experiments. ** *p* < 0.01, *** *p* < 0.001.

To further study the effects of Treg on hAMSC, we investigated the differentiation and paracrine function of hAMSC *in vitro*. Astonishingly, we found that after Treg treatment, the differentiation such as the adipogenic ability of hAMSC was enhanced (Figure 2A). As a result in Figure 1 indicated HGF and SDF-1 expression may be the cause for hAMSC’s protection, we analyzed the cultured supernatants of two groups through ELISA. The results showed that Treg treatment contributed to the upregulation of paracrine production of hAMSC (Figure 2B).

### Human Amniotic Mesenchymal Stem Cell Secreted Hepatocyte Growth Factor Participated in Resisting Liver Cirrhosis Damage

**FIGURE 3:**
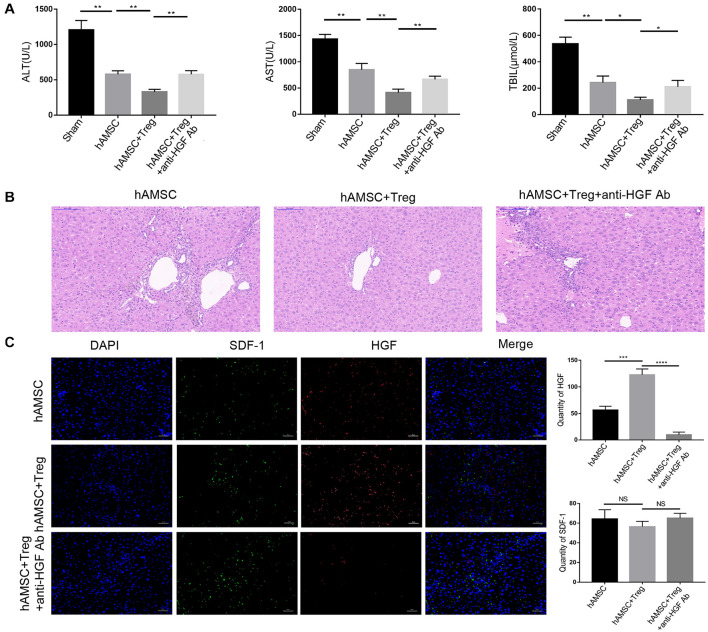
PBS, hAMSC, hAMSC + Treg, and hAMSC + Treg + anti-HGF Ab were injected into mice through the tail vein, respectively. **(A)** Level of ALT, AST, and TBIL of four groups. **(B)** Representative images of liver tissue [hematoxylin and eosin (HE) staining] by light microscopy in three groups. **(C)** Representative fluorescent images for SDF-1 and HGF in three groups were visualized by fluorescence microscopy, and DAPI was used to mark nuclei. The result is representative of three independent experiments. Data were mean ± SD of three independent experiments. * *p* < 0.05, ** *p* < 0.01, *** *p* < 0.001.

As HGF is important for liver regenesis, and Figure 2 had indicated that Treg successfully improved the hAMSC produced HGF. So we used an anti-HGF monoclonal antibody (25 ⋅ g/kg) to abolish HGF *in vivo*. Mice were divided into three groups and each group contained four mice. The addition of anti-HGF Ab decreased the protective effect of hAMSC and Treg co-transfer and it significantly up-regulated the liver enzyme levels (Figure 3A). And liver biopsy in Figure 3B showed that anti-HGF Ab weakened the anti-cirrhosis function of hAMSC. Besides, confocal fluorescence microscopy showed that after the addition of the anti-HGF Ab, the expression of HGF was significantly reduced. At the same time, the expression of SDF-1 did not change significantly (Figure 3C), which indicated that HGF participated in repairing liver injury during the protective ability of hAMSC and Treg.

### TGF-β Significantly Affected the Activation and Function of Human Amniotic Mesenchymal Stem Cell

**FIGURE 4:**
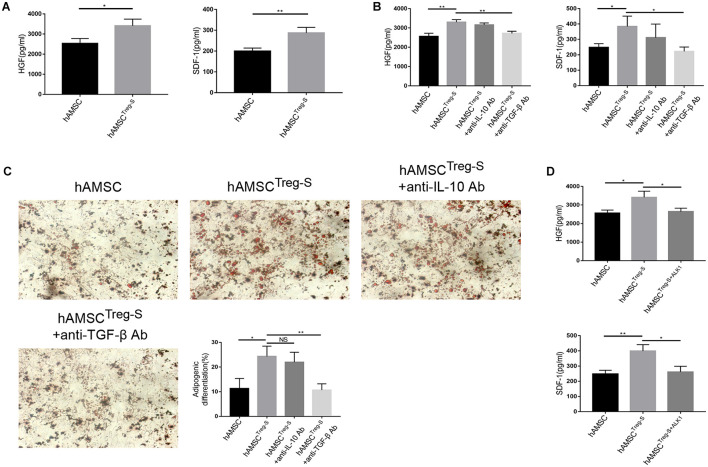
hAMSC was treated with supernatant of Treg, anti-IL-10 Ab and anti-TGF-β Ab *in vitro*. **(A)** Level of HGF and SDF-1 of hAMSC and hAMSC^*Treg–S*^. **(B)** Level of HGF and SDF-1 in hAMSC, hAMSC^*Treg–S*^, hAMSC^*Treg–S*^ with anti-IL-10 Ab and hAMSC^*Treg–S*^ with anti-TGF-β Ab. **(C)** Representative images of hAMSC’s adipogenic differentiation in four groups. **(D)** Level of HGF and SDF-1 of hAMSC, hAMSC^*Treg–S*^, and hAMSC^*Treg–S*^ with ALK1. The result is representative of three independent experiments. Data were mean ± SD of three independent experiments. * *p* < 0.05, ** *p* < 0.01.

As Treg improved the function of hAMSC *in vitro*, we hypothesized that Treg regulated hAMSC in an acellular contact manner. Treg was cultured in a non-cytokine environment for two days. The supernatant was collected and added into the hAMSC culture system (hereafter referred to as hAMSC^*Treg–S*^). As we expected, HGF and SDF-1 were greatly upregulated with the addition of the Treg’s supernatant (Figure 4A). TGF-β and IL-10 are the two most important cytokines secreted by Treg (Nono et al., 2020; Bertolini et al., 2021). We added anti-IL-10 monoclonal antibody and anti-TGF-β monoclonal antibody, respectively, and found that only anti-TGF-β Ab down-regulated both the HGF and SDF-1 expression *in vitro* (Figure 4B). We also tested the cell differentiation, which showed the supernatant of Treg improved the suppressive ability of hAMSC and anti-TGF-β Ab weakened the differentiation ability and decreased the number of adipose cells (Figure 4C). Finally, we used ALK1, the neutralizer of the TGF-β type I receptor, to verify the effect of TGF-β on hAMSC (Salmon et al., 2020). Data verified that HGF and SDF-1 secretion by hAMSC^*Treg–S*^ after ALK1 treatment is significantly reduced (Figure 4D).

### Indoleamine 2,3-Dioxygenase Signaling Is Involved in Human Amniotic Mesenchymal Stem Cell^*Treg–S*^ Function and Differentiation Ability

**FIGURE 5:**
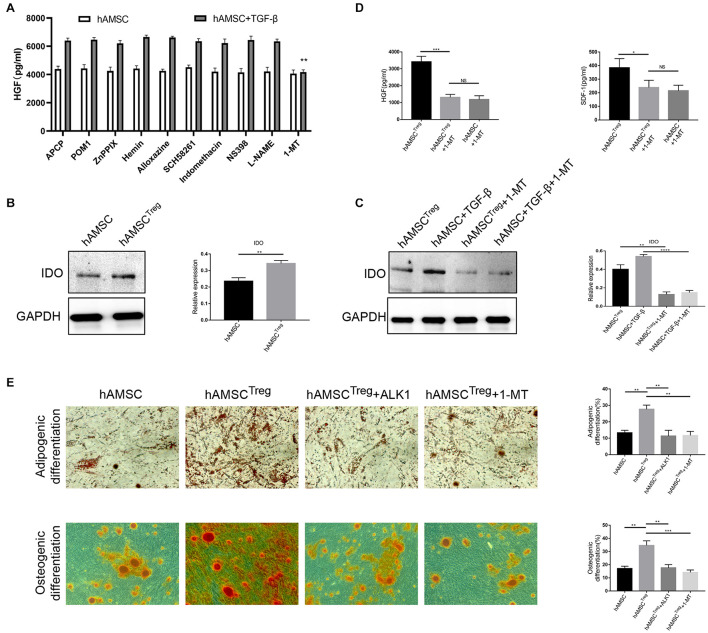
Multiple signal neutralizers were added to the culture system and 1-MT was proved to be effective. **(A)** Level of HGF in hAMSC’s supernatant after adding multiple signal neutralizers. APCP: CD73 inhibitor; POM1: CD39 inhibitor; ZnPPIX: HO-1 inhibitor; Hemin: HO-1 inducer; ALLoxazine: adenosine receptor inhibitor; SCH58261: adenosine A2A receptor inhibitor; Indomethacin: COX inhibitor; NS398: COX-2 inhibitor; L-NAME: NOS inhibitor; 1-MT: IDO inhibitor. **(B)** IDO expression of PBS treated hAMSC and hAMSC^*Treg–S*^ by western blot and the relative expression calculated by IDO versus GAPDH through Image J software. **(C)** IDO expression of hAMSC^*Treg–S*^, TGF-β, hAMSC^*Treg–S*^ with 1-MT, and TGF-β with 1-MT by western blot and the relative expression calculated by IDO versus GAPDH through Image J software. **(D)** Level of HGF and SDF-1 in hAMSC, hAMSC^*Treg–S*^, and hAMSC^*Treg–S*^ with 1-MT. **(E)** Adipogenic and osteogenic differentiation of hAMSC, hAMSC^*Treg–S*^, hAMSC^*Treg–S*^ with ALK1, hAMSC^*Treg–S*^ with 1-MT. The result is representative of three independent experiments. Data were mean ± SD of three independent experiments. * *p* < 0.05, ** *p* < 0.01, *** *p* < 0.001.

To further understand the mechanism of TGF-β in regulating hAMSC function, multiple signal neutralizers were added to the culture system. Among all the neutralizers, only 1-MT, the suppressor of IDO signaling, reduced HGF expression by hAMSC (Figure 5A). After that, we used western blot to prove that the IDO signal was upregulated in TGF-β signaling, and 1-MT successfully down-regulated the IDO expression (Figures 5B,C). We also tested the HGF, SDF-1 expression with the presence of 1-MT, which showed that HGF and SDF-1 were also decreased by 1-MT (Figure 5D). To further investigate the differentiation ability of hAMSC^*Treg–S*^, adipogenic differentiation and osteogenic differentiation were performed. The results showed that both ALK1 and 1-MT significantly reduced the differentiation ability of hAMSC^*Treg–S*^ either in the adipogenic or osteogenic environment (Figure 5E).

### Treg Improved the Function of Human Amniotic Mesenchymal Stem Cell in Reducing Liver Cirrhosis Through Regulating Indoleamine 2,3-Dioxygenase Signaling

**FIGURE 6:**
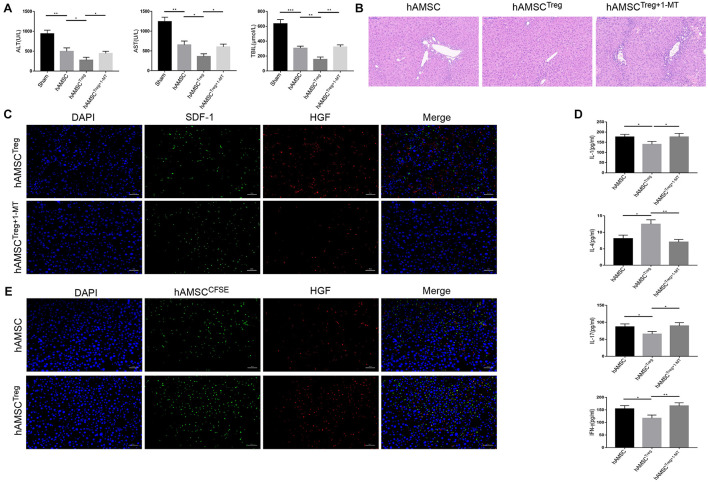
PBS, hAMSC, hAMSC^*Treg*^, and hAMSC^*Treg+*1–MT^ were injected into mice through the tail vein, respectively. **(A)** Level of ALT, AST, and TBIL of four groups. **(B)** Representative images of liver tissue [hematoxylin and eosin (HE) staining] by light microscopy in hAMSC, hAMSC^*Treg*^, and hAMSC^*Treg+*1–MT^ group. **(C)** Representative fluorescent images for SDF-1 and HGF in hAMSC^*Treg*^ and hAMSC^*Treg+*1–MT^ group were visualized by fluorescence microscopy, and DAPI was used to mark nuclei. **(D)** Level of IL-1, IL-4, IL-17, and IFN-γ in hAMSC, hAMSC^*Treg*^, and hAMSC^*Treg+*1–MT^ group. **(E)** Representative fluorescent images for hAMSC and HGF were visualized by fluorescence microscopy, and DAPI was used to mark nuclei. The result is representative of three independent experiments. Data were mean ± SD of three independent experiments. * *p* < 0.05, ** *p* < 0.01, *** *p* < 0.001.

Finally, we detected the mechanism of Treg treated hAMSC in protecting liver cirrhosis *in vivo*. The murine liver cirrhosis model was established as described above, and hAMSC, Treg pre-treated hAMSC (mixed co-cultivation, hereinafter referred to as hAMSC ^*Treg*^) as well as Treg and 1-MT pre-treated hAMSC (hereinafter referred to as hAMSC^*Treg+*1–MT^) were injected into mice, respectively. Each group contained four mice. The results showed that hAMSC^*Treg*^ presented a better effect on reducing liver enzymes and total bilirubin. But the effect was recalled after the addition of 1-MT (Figure 6A). Hematoxylin-eosin also showed that the addition of 1-MT greatly weakened the impact of hAMSC^*Treg*^ against liver fibrosis (Figure 6B). Confocal fluorescence microscopy showed that after the addition of 1-MT, the expression of HGF and SDF-1 in hAMSC^*Treg*^ were significantly reduced (Figure 6C). We also tested the expression of cytokines in the liver, which showed that the addition of 1-MT increased the pro-inflammatory cytokine expression such as IL-1, IL-17, IFN-γ and decreased the expression of IL-4 comparing with hAMSC^*Treg*^ (Figure 6D). To verify that the injected hAMSC did reach the liver and secreted HGF, we labeled the hAMSC and hAMSC^*Treg*^ with CFSE dye and injected it into the tail vein. The results showed that comparing with hAMSC, more amounts of hAMSC^*Treg*^ were accumulated in the liver and the expression of HGF also increased under the fluorescence microscope (Figure 6E).

## Discussion

Mesenchymal stem cells have presented therapeutic potential in various diseases through immunomodulatory and tissue repair functions. Recent studies showed that MSCs are functional in many liver diseases such as liver fibrosis, cirrhosis, and liver failure. In clinical studies registered on www.ClinicalTrials.gov, trials had been taken to treat liver failure, liver transplantation, liver fibrosis, autoimmune liver disease, and so on through MSCs transferring. Studies have reported that mesenchymal stem cells secrete various cytokines, chemokines, growth factors, and exosomes, which help indirect and remote tissue repair. It is important that mesenchymal stem cells regulate nutritional factors and indirectly mediate anti-apoptosis, anti-oxidation, anti-fibrosis, angiogenesis and immunosuppressive effects (Wang et al., 2014) and mesenchymal stem cells can synergistically improve liver fibrosis with induced M2 macrophages (Watanabe et al., 2019). However, reports showed limited progress of MSCs in anti-cirrhosis in the liver, and the reason may be related to severe inflammation (Hu et al., 2020). Many inflammatory factors were expressed in the stage of liver cirrhosis, which aggravated the damage during liver cirrhosis. Treg was proved to reduce inflammation in liver disease by suppressing immune status. We thought to use hAMSC to repair tissue while reducing inflammation through reinfusion of Treg.

As we expected, Treg inhibited inflammation during cirrhosis, but surprisingly, improve the function of AMSCs. Co-transfer of Treg downregulated the injury and delayed the cirrhosis process of the liver. Depletion of HGF abolished the treatment effect of co-transfer, indicating that Treg improved the therapeutic impact through improving the cytokine expression ability of hAMSC. *In vitro* experiment surprisingly showed that Treg improved the HGF and SDF-1 expression of hAMSC. As the supernatant of Treg showed a similar result as Treg itself, we hypotheses that Treg expressed pro-inflammatory cytokines such as TGF-β and IL-10 may be the key for Treg’s regulation of hAMSC. Using the antibody of TGF-β or IL-10, we found that TGF-β Ab suppressed the upregulation of HGF in supernatant treated hAMSC. Besides, further mechanism studies showed that TGF-β improved hAMSC’s function through regulating the IDO signaling.

TGF-β played an important role in maintaining normal human development and homeostasis. It binds to a membrane receptor with a cytoplasmic serine/threonine kinase domain. IDO had been proved to have an indispensable effect on the immune regulation ability of human MSC (Munn and Mellor, 2013; Su et al., 2014). It promoted metabolic immune regulation by catalyzing the oxidative catabolism of the essential amino acid tryptophan (TRP) in the kynurenine (KYN) pathway (Munn and Mellor, 2013). Studies have reported that TGF-β can up-regulate the expression of IDO in cultured cells *in vitro*. And IDO gene silencing technology can eliminate the increased IDO expression of TGF-β (Fallarino et al., 2005; Pallotta et al., 2011). Studies have also reported that the expression of TGF-β is positively correlated with the expression of IDO in the chorionic villi and decidua tissues of normal pregnant women (Liu et al., 2017). In our research, we found that Treg promoted the IDO signaling pathway of hAMSC by secreting TGF-β, thereby secreting more cytokines such as HGF and SDF-1 and reducing the damage of liver cirrhosis.

MSCs mainly inhibit liver fibrosis by differentiating into hepatocyte-like cells (Dai et al., 2009), secreting nutritional factors, expressing a variety of soluble factors to regulate the proliferation and function of a variety of immune cells, as well as antioxidant activity (Eom et al., 2015). However, as liver cirrhosis is an end-stage organic disease, studies have confirmed that MSCs have a poor therapeutic effect. Lipopolysaccharide (LPS) or endotoxin promotes systemic inflammation by activating TLR-2 and TLR-4 dependent pathways and promoting cytokines in large quantities, thereby accelerating the progression of liver cirrhosis (Wilde and Katsounas, 2019). Treg is functional in suppressing T and other immune cells’ activation and thereby inhibiting inflammation. Treg has a comprehensive immune regulation function. First, Treg secretes heterogeneous cytokines, such as IL-10, IL-35, and TGF-β, which inhibit the pro-inflammatory response. Secondly, the cytotoxic T lymphocyte antigen-4(CTLA-4) expressed by Treg cells can impair the maturation of APC. Finally, Treg plays a role through immune checkpoint molecules ICOS and LAG-3 and immunosuppressive metabolites CD39, CD73 and IDO (Sharabi et al., 2018). Although MSCs themselves can suppress immunity, and their functions are not as comprehensive as Treg. More importantly, we discovered for the first time that Treg could improve the function of hAMSC. And TGF-β-IDO signaling may be the possible mechanism, but further research is necessary to analyze.

When selecting the type of MSCs at the beginning of the experiment, we chose hAMSC because of its rich source, limited immunogenicity, and negligible ethical issues (Lobo et al., 2016; Shi et al., 2017; Silini et al., 2017). Human amniotic mesenchymal stem cell has many applications. For example, it can secrete a variety of cytokines to promote angiogenesis and bone formation. Therefore it is expected to be an alternative method for bone tissue regeneration. Our research showed that co-infusion of hAMSC and Treg might provide a promising approach for treating liver cirrhosis.

## Data Availability Statement

The original contributions presented in the study are included in the article/supplementary material, further inquiries can be directed to the corresponding author.

## Ethics Statement

The animal study was reviewed and approved by IACUC-2008036.

## Author Contributions

ZD, JRZ, and XM designed the experiments. JG, XL, QS, JL, CY, GH, and JZ performed the experiments, analyzed the data, and wrote the manuscript. YX designed the overall concept, analyzed the data, and wrote the manuscript. All authors contributed to the article and approved the submitted version.

## Conflict of Interest

The authors declare that the research was conducted in the absence of any commercial or financial relationships that could be construed as a potential conflict of interest.

## Publisher’s Note

All claims expressed in this article are solely those of the authors and do not necessarily represent those of their affiliated organizations, or those of the publisher, the editors and the reviewers. Any product that may be evaluated in this article, or claim that may be made by its manufacturer, is not guaranteed or endorsed by the publisher.
